# Impaired Self-Awareness and Denial During the Postacute Phases After Moderate to Severe Traumatic Brain Injury

**DOI:** 10.3389/fpsyg.2020.01569

**Published:** 2020-07-16

**Authors:** George P. Prigatano, Mark Sherer

**Affiliations:** ^1^Department of Clinical Neuropsychology, Barrow Neurological Institute, Phoenix, AZ, United States; ^2^TIRR Memorial Hermann, Houston, TX, United States

**Keywords:** anosognosia, impaired self-awareness, denial, traumatic brain injury, intervention

## Abstract

While a number of empirical studies have appeared on impaired self-awareness (ISA) after traumatic brain injury (TBI) over the last 20 years, the relative role of denial (as a psychological method of coping) has typically not been addressed in these studies. We propose that this failure has limited our understanding of how ISA and denial differentially affect efforts to rehabilitate persons with TBI. In this selective review paper, we summarize early findings in the field and integrate those findings with more recent observations (i.e., 1999–2019). We believe that this synthesis of information and expert clinical opinion will inform future research on ISA and denial as well as approaches to rehabilitation for persons with TBI.

## Introduction

Early efforts at modern day neuropsychological rehabilitation of postacute adults with moderate to severe traumatic brain injury (TBI) identified impaired self-awareness (ISA) as an important barrier to successful rehabilitation outcome, particularly when the goal was to return the person to a productive life style (Prigatano et al., 1984). Subsequent research has attempted to explicate these disturbances by identifying their etiology, prevalence, and predictive value for the process and outcome of various forms of rehabilitation after moderate to severe TBI (Prigatano, 2005; Sherer and Fleming, 2014, 2019). A persistent issue that has overshadowed this research is the failure to study the impact that denial may have on research findings regarding ISA after TBI. Denial is a psychological method of coping that can also affect accuracy of self-perceptions for some patients following moderate to severe TBI. For example, Niemeier et al. (2016) reported that patients with a premorbid history of substance use disorder had greater ISA than those without a history of substance use disorder. While they related their findings to possible greater executive dysfunction prior to the TBI in the substance use disorder group, they did not determine whether denial within the substance use disorder prior to the TBI contributed to postinjury underreporting of deficits. It is well recognized that patients with substance use disorder often do not report their substance use to be a problem, and this appears to be a form of denial that is a major barrier to treatment (Rogers et al., 2019).

Understanding the potential role of denial in contributing to patients’ underestimation of the extent of cognitive and behavioral problems after moderate to severe TBI is important for at least two major reasons. First, such understanding may explain why various neurological and neuropsychological markers of severity of TBI are typically only moderately correlated with measures of severity of ISA. Second, greater understanding of the role of denial may provide further insights into how to best engage this heterogeneous patient group with various treatment/rehabilitation activities aimed at improving their overall recovery of function and long-term adaptation after moderate to severe TBI.

This selective review summarized early empirical findings that provide a context for integrating more recent clinical and research observations regarding ISA and denial of disability (DD) in postacute TBI. Before describing the methodology for this review, it is important to revisit the definitions of ISA and DD.

### Definition of ISA

Prigatano and Schacter (1991) defined self-awareness as “…the capacity to perceive the ‘self’ in relatively ‘objective’ terms while maintaining a sense of subjectivity.” They go on to note: “self-awareness or awareness of higher cerebral functions thus involves an interaction of ‘thoughts’ and ‘feelings.” It is not a purely cognitive function (Prigatano, 2014, 2020), and for this reason, it is difficult to measure. Based on this proposition, ISA following moderate to severe TBI is defined as a failure to experience (as assessed via subjective reports) a disturbance in higher integrative brain functions due to a disruption or damage to regions of the brain that are important for the normal performance of those higher integrative brain functions (Prigatano, 2020). This definition is based on Gabriel Anton’s early observations on unawareness of cortical blindness (Anton, 1898; Forstl et al., 1993). Since there is a lack of subjective experience of impairments due to failure to integrate “feelings and thinking,” ISA is not associated with an emotional reaction *per se*. In fact, emotional reactivity in these patients may be greatly diminished or absent altogether. Consequently, affected patients are often described as “neutral” or “perplexed” when given feedback concerning their neuropsychological impairments and associated behavioral disturbances (Prigatano and Klonoff, 1998). They appear “bland,” “apathetic,” or “indifferent” to an impairment that is obvious to an observing clinician (Prigatano, 2020).

Impaired self-awareness, like any other disruption of brain function, can be grossly classified according to levels of severity such as “mild,” “moderate,” and “severe” (Prigatano, 2010). Persons with TBI may have different levels of ISA for different functional domains (Prigatano, 2014). Low levels of ISA may have little or no effect on functional outcomes (Sherer et al., 2003a), while clinically significant ISA affects both functional outcome and the ability to engage in treatments after moderate to severe TBI.

Clinically significant ISA can be operationally defined as a disturbance in self-awareness that results in a person making behavioral choices and experiencing reactions that negatively impact their functioning in everyday life, including engaging in necessary treatment/rehabilitation activities, self-care activities, maintaining interpersonal relationships, and obtaining and sustaining a productive life. Clinicians often make a judgment about the presence or absence of clinically significant ISA based on multiple sources of information. This often begins with a patient’s self-description in the clinical interview and their responses to questionnaires regarding levels of functioning. However, observations of the patient’s behavior in interpersonal situations (such as their reaction to rehabilitation activities, responses to failures on neuropsychological tests, and explanations for repeated failures at work) often facilitate reliable judgments regarding whether a clinically significant level of ISA is present. Unfortunately, only a few investigations have attempted to derive empirically based cutoffs for clinically significant ISA (Sherer et al., 2003a).

### Definition of Denial of Disability

Denial has classically been defined as one of the defense mechanisms. The Diagnostic and Statistical Manual of Mental Disorders IV published by the American Psychiatric Association (American Psychiatric Association, 1994) has this to say about defense mechanisms and denial. A defense mechanism is an “automatic psychological process that protects the individual against anxiety and from awareness of internal or external stressors or changes. Defense mechanisms mediate the individual’s reaction to emotional conflicts and to external stressors. Some defense mechanisms (e.g., projection, splitting, and acting out) are almost invariably maladaptive. Others, such as suppression and denial, may be either maladaptive or adaptive, depending on their severity, their inflexibility, and the context in which they occur.” (p. 765) Lazarus (Lazarus, 1983) emphasizes that denial is a “negation of something in word or act, or more properly, both, since thoughts and actions are apt to be conjoined in any defense process” (p. 19). He goes on to say, “in speaking of the denial process, one is immediately faced with multiple ambiguities. One of the most common sources of confusion is the equation of denial with *avoidance*” (p. 10). He notes that denial might be reflected in failure to pay attention to a realistic threat, or it might be reflected in avoidance of any thought or discussion of a problem a person faces that seems overwhelming to them. Avoidance may also be associated with simply not knowing how to deal with a problem that is in fact recognized (and verbally admitted to) by the person. There is not a single behavioral “marker” of denial. Yet, what is agreed upon is that as a defense mechanism, denial reflects the process by which the individual attempts to deal with emotional conflicts in the presence of external stressors. Thus, denial is a complex cognitive–behavioral–emotional reaction.

Various behavioral/emotional responses appear to be associated with denial of neuropsychological impairment with associated disability after moderate to severe TBI (Prigatano and Klonoff, 1998). Such responses may include disavowing the potential importance of a problem that is recognized by the person (e.g., yes, I have a memory problem but it does not really affect my work), an angry reaction when faced with a performance failure (e.g., my day to day memory really is good, your tests of memory are unfair or do not really measure memory capacity in everyday life), and avoidance of discussing the impairment and associated disability (e.g., I am tired about talking about my memory impairment. It is not helping me. Leave me alone and stop discussing it!). In each of the behavioral reactions associated with denial of impairments and related DD, there is an attempt by the patient to dissuade or discourage any further dialog regarding a neuropsychological impairment and associated disability. The examining clinician senses that the person wants to stop talking about the problems revealed by neuropsychological examination or manifested during rehabilitation activities (Prigatano, 2020). Family members often comment that the patient will make life difficult for them at home if they keep discussing the problems that the patient has in day-to-day functioning that appear clearly related to the TBI.

Given these considerations, denial of disability (DD) is defined as an emotional reaction (often automatic or non-conscious in nature) that attempts to keep anxiety-provoking feelings and thoughts from reaching awareness. We propose that common markers of DD are disavowal of the importance of a partially recognized problem, angry reactions when discussing that problem, avoidance of discussing the problem, and, perhaps most importantly, any attempt that discourages others from talking to the person about “the problem.”

In light of these definitions it is important to restate that ISA is not generally associated with emotional reactions such as anxiety. In contrast, DD is characteristically associated with emotional reactions particularly anxiety and is an attempt to avoid the subjective experience of anxiety.

To provide guidance to clinicians and researchers, the authors conducted a selective review of articles published in the past 20 years that make significant contributions to our knowledge regarding ISA and DD. Information from these articles was combined with the clinical expertise of the authors that is based on several combined decades of experience in addressing ISA and DD in the rehabilitation of persons with TBI. Since there were relatively few papers devoted to understanding and measuring denial in the population of persons with TBI, selected papers that dealt with the topic of denial in other patient groups were also reviewed in the hope of applying insights from those papers to the study of DD in patients with TBI. Our overall goals were to provide guidance for clinical care and to specify a research agenda to guide future research regarding these important topics.

## Methods

The authors sought to address study goals by conducting a selective review of the literature and integrating evidence derived from this review with expert clinical opinion. In order to survey the recent literature on ISA and DD, three methods were used. First, a search of the literature on ISA and DD after TBI published from 1/1/1999 to 11/08/2019 was conducted using the PubMed database. All search terms were “exploded.” The strategy for this search is provided in Table 1. Abstracts were retained primarily based on the authors’ perception that they contributed to the study goals listed above. Additional abstract review criteria are provided in Table 2. The articles associated with retained abstracts were carefully reviewed, and those that made contributions to this study were retained. Second, the authors viewed the reference lists from retained articles to search for additional papers that could contribute to this project. Third, authors identified articles from their own knowledge of the relevant literature to include for this review.

**TABLE 1:** Search terms and strategy for the literature search on awareness and denial after TBI (1999–2019).

**Search Terms**
Awareness
Self-awareness
Impaired awareness
Metacognition
Denial
Traumatic brain injuries
Brain injuries, traumatic

*Traumatic brain injuries AND Denial OR awareness OR metacognition. From 01/01/2009 to 11/08/2019. TBI, traumatic brain injury.*

**TABLE 2:** Inclusion and exclusion criteria for abstract reviews.

**Inclusion criteria**
□ Article available in English
□ Participants include at least one person with TBI
□ 80% of cohort has TBI or there is a TBI subanalysis
□ Article addresses issues regarding self-awareness or denial such as:
∘ Definition of self-awareness and/or denial
∘ Incidence of impaired self-awareness and/or denial
∘ Assessment of impaired self-awareness and/or denial
∘ Neurologic and behavioral correlates of self-awareness and/or denial
∘ Change of self-awareness and/or denial over time
∘ Comparison of impaired self-awareness and denial
**Exclusion criteria**
□ Study participants are in a Disorder of Consciousness
□ Study participants are in the Post-traumatic Confusional State
□ Study findings are not considered reliable due to bias or poor design

*TBI, traumatic brain injury.*

All retained and subsequently identified articles were carefully read and summarized by one of the authors. While evidence was derived from the retained articles from the literature search and additional identified articles, consistent with the planned approach for this study, evidence was not summarized in evidence tables. Rather, evidence was described in *Results* and integrated with expert clinical opinion to produce the observations provided in section “Discussion.”

## Results

The literature search returned 247 unique abstracts. After review of these abstracts and associated articles, 88 articles were retained to contribute to the paper. Twenty-one additional articles were identified by reviewing reference lists from the retained articles and including articles based on direct knowledge of the authors.

### The Measurement of ISA After TBI

At least four methods of quantifying ISA have been used in research on impaired awareness. The most commonly used of these approaches is the discrepancy method. In this approach, the person with TBI rates his/her ability to perform various cognitive, neurobehavioral, and functional tasks. A second person also rates these abilities for the person with injury. The discrepancy (commonly measured as the value obtained by subtracting the other observer rating from the self-rating) indicates severity of ISA. Other ratings are usually obtained from a treating clinician in the early postinjury period and from a family member/close other in the postacute period. ISA measured in this manner is referred to as metacognitive awareness as the person with injury is required to think about and assess his/her own cognitive abilities.

Reviews of the ISA literature have indicated that the Patient Competency Rating Scale and the Awareness Questionnaire are the most commonly used and best validated discrepancy measures of ISA (Smeets et al., 2012; Al Banna et al., 2016). Prigatano et al. (1986) developed the Patient Competency Rating Scale (PCRS) as a guide to the clinical interview with patients. Patients were asked to rate their level of difficulty performing a variety of tasks including activities of daily living, their ability to understand new instructions, their ability to perform memory tasks, and their ability to control emotions and socially interact with others. A significant other (typically a family member) independently rated the patient’s competency level on each task. A discrepancy score was calculated, and it was noted that persons with a history of postacute (i.e., at least 1 year post) moderate to severe TBI often underreported their levels of difficulty performing these tasks compared to significant others’ ratings (Prigatano et al., 1990; Prigatano, 1996). Thus, when the patient’s total rating score (which can range from 30 to 150 points) was higher than the relative’s total rating score, behavioral evidence suggested that the patient had ISA. The question that emerged, however, was what magnitude of discrepancy constitutes a reliable behavioral maker of clinically significant ISA? While early work with this scale suggested that a 9- or 10-point discrepancy score of overestimating abilities (or conversely underreporting levels of difficulty) compared to significant other’s reports might signal a clinically significant ISA (Prigatano et al., 1998), more recent work has suggested that a 20-point discrepancy score may provide the clearest classification of patients with clinically significant ISA after moderate to severe TBI (Bivona et al., 2019).

It should be emphasized, however, that ISA is a disturbance of subjective or phenomenological experience (Prigatano and Schacter, 1991; Prigatano, 2010, 2020). Thus, any behavioral rating scale always measures ISA “indirectly.” The discrepancy ratings/scores suggest that the patient is experiencing some compromise in their subjective appraisal of their disturbed neuropsychological and related functions. In this regard, another important point has to be made. Patients who underreport their competencies compared to relative’s reports (i.e., state they are functioning at a significantly lower level then what relatives or significant others report) are not considered to demonstrate ISA as a neurologically based disturbance. Later in this paper, empirical findings that support this proposition will be discussed.

The Awareness Questionnaire (AQ) was developed by Sherer et al. (1998) and consists of 17 items as compared to 30 items for the PCRS. Regarding item content, PCRS items focus on functional abilities, while AQ items focus on areas of impairment often seen after TBI. Items for both measures are rated on 5-point Likert-type scales. Anchors for the PCRS indicate the amount of difficulty that the respondent has in performing a task and range from “can’t do” to “can do with ease.” Anchors for the AQ indicate how the respondent’s current abilities compare to his/her abilities prior to the injury and range from “much worse” to “much better.” Thus, respondents on the AQ can rate themselves as having more intact sensory/motor, cognitive, and behavioral/affective abilities after TBI than before. This pattern is not rare and is essentially always associated with severe ISA.

While the PCRS and AQ are similar in many ways, scores from the two measures are only moderately correlated. Sherer et al. (2003a) found that PCRS patient self-ratings correlated 0.50 with AQ patient self-ratings, PCRS family ratings correlated 0.62 with AQ family ratings, and PCRS clinician ratings correlated 0.69 with AQ clinician ratings.

The second most commonly used method to assess ISA after TBI is clinician rating based on a structured clinical interview. The most commonly used and best validated measure of this type is the Self-Awareness of Deficits Interview develop by Fleming et al. (1996). For this measure, the clinician is provided with initial questions to ask the patient such as “Are you any different now compared to what you were like before your accident?” The interviewer has a suggested list of prompts to be used to follow-up on the patient’s initial responses. Following the interview, the interviewer rates the patient on self-awareness of impairments, awareness of functional implications, and ability to set realistic goals. Each area is rated from 0 to 3, with higher scores indicating greater impairment of self-awareness. While one could be concerned that Self-Awareness of Deficits Interview ratings might vary depending on the person doing the interview, in a study of 80 outpatients with TBI, Ownsworth et al. (2019) found 80–84% agreement in classification of severity of ISA between the SADI and the AQ. This finding offers support for the validity of both measures. In addition, it should be noted that methods for assessing denial after TBI depend on structured interview.

A third less frequently used method for assessing ISA is comparison of self-perceptions to scores on cognitive tests. This method can be used to compare self-perceived cognitive abilities to a comprehensive cognitive assessment (Anderson and Tranel, 1989) or to compare the patient’s estimation of his/her ability on a single test to their actual performance (Fischer et al., 2004). Note that when a single or small number of tests are used, repeated trials of the patient predicting his/her performance and then observing the actual performance can be used as an intervention to improve ISA (Rebmann and Hannon, 1995).

A final approach to measuring ISA assesses online awareness. In this method, the patient is observed to perform a test of sustained attention (McAvinue et al., 2005) or a multistep functional task (Hart et al., 1998). As persons with TBI perform these tasks, they periodically make mistakes. An observer tallies the number of errors and notes whether the respondent noticed the error and attempted to correct the error. A greater number of unnoticed errors indicate poorer online awareness. There is limited evidence regarding the extent to which these self-prediction and online awareness measures relate to the broader phenomenon of ISA that has been shown to affect patient’s engagement in treatment and eventual outcomes.

### Incidence of Clinically Significant ISA in Postacute Patients With a History of Moderate to Severe TBI

In an early study, (Oddy et al., 1985) reported that 40% of postacute TBI patients “refused to admit to difficulties.” Prigatano and Altman (1990), using the PCRS to measure ISA, reported that in a sample 64 postacute patients with mild to severe TBI, 25% reported greater functional competency compared to their relative’s report. Studying a Spanish sample of persons with a history of mild to severe TBI, Prigatano et al. (1998) reported that 40% “rated themselves higher than their relatives rated them on the PCRS” by 5 points or more. Whether a 5-point disparity on the PCRS captures clinically significant ISA is questionable. After following patients with a history of severe TBI and persistent and severe ISA over a 20- to 25-year period, it was suggested that a disparity of a least 20 points on the PCRS would provide one marker of clinically significant ISA (Prigatano, 2014). Using this criterion (Bivona et al., 2019) recently reported that 20% of postacute patients with a history of moderate to severe TBI meet this criteria of severe ISA that appears to be clinically significant. In a study from Netherlands (Smeets et al., 2017), 78 patients with a history of either TBI or cerebral vascular accidents (CVAs) were classified as overestimating, underestimating, or having accurate estimation of their competencies using the PCRS. The overestimating group (i.e., the group thought to have ISA) had an average disparity score of + 18.3 points. Twenty out of the 78 patients were in this “overestimation group,” suggesting that 25.6% appeared to show clinically significant ISA.

Thus, studying postacute patients with TBI using the PCRS in the United States, Spain, Italy, and Netherlands, it appears that 20–25% of patients with moderate to severe TBI can be classified as having clinically significant ISA. Studies using measures other than the PCRS have produced similar findings. In a study of patients with TBI who were undergoing inpatient rehabilitation (Sherer et al., 2003a) found that patient–clinician discrepancies of 20–29 on the AQ were associated with a decrease in the likelihood of a favorable functional at discharge. Discrepancies >29 for the AQ strongly predicted an unfavorable outcome. Fewer than 25% of participants fell in the poorest outcome categories for ISA. Using a different methodology, (Ownsworth et al., 2019) found that 12% of a cohort of 80 patients with moderate/severe TBI fell in their greatest severity of ISA category when assessed with the SADI, while 26% fell in the worst ISA category when assessed with the AQ.

### Neurological Correlates of ISA in Patients With a History of Moderate to Severe TBI

In an attempt to relate ISA to traditional indicators of severity of TBI, (Prigatano and Altman, 1990) reported that ISA was marginally (but non-significantly) related to the patients’ admitting Glasgow Coma Scale (GCS) score. Prigatano et al. (1998) later reported that postacute patients with TBI who overestimate their behavior competency on the PCRS have lower admitting GCS scores (*r* = −0.39; *p* = 0.05) and longer periods of PTA (*r* = +0.41, *p* < 0.05). Sveen et al. (2015) replicated the GCS findings with a Norwegian version of the PCRS. Sandhaug et al. (2012) reported the ISA of cognitive difficulties, as measured by selected items of the PCRS, was predicted by acute GCS scores and duration of PTA. Sherer et al. (2003b) found significant although modest correlations of GCS scores and duration of coma (measured as time to follow commands) with degree of ISA.

Other investigators, however, have not found that the GCS score or the length of PTA correlates with ISA (Ciurli et al., 2011). Failure to replicate these findings might, in part, be explained by the failure to separate patients with TBI who overestimate their cognitive abilities secondary to denial versus altered self-awareness due to underlying brain dysfunction (i.e., ISA).

Prigatano and Altman (1990) initially reported that the number of neuroradiographic abnormalities identified on CT or MRI scans were related to the presence of ISA in patients with TBI. Sherer et al. (2005) replicated these findings. Later research, however, has failed to demonstrate that specific measures of ISA are related to the degree of structural brain abnormalities following TBI (Ham et al., 2014). Rather, different forms of ISA appear to be related to specific areas of brain function/dysfunction. For example, Schmitz et al. (2006) reported that good self-awareness, as measured by the PCRS after severe TBI, was related to increase signal change of the right anterior dorsal prefrontal cortex when performing a self-reflection task. Using a self-monitoring task to detect errors, patients with TBI who had “low performance monitoring” and underestimated their disability showed broad attentional deficits. This group had greater resting functional connectivity abnormalities involving frontal and parietal regions of the brain (Ham et al., 2014). These findings and others (Spikman and van der Naalt, 2010) began to reinforce the idea that underlying frontal lobe pathology may greatly contribute to ISA in postacute patients with TBI. If this is the case, neuropsychological test scores sensitive to frontal lobe dysfunction might correlated with ISA measures. They would not be expected to correlate with measures of denial of disability (DD).

### Neuropsychological Correlates of ISA in Patients With a History of Moderate to Severe TBI

Prigatano and Altman (1990) studied the relationship of ISA to various neuropsychological measures. Using measures of intelligence, memory and novel or abstract reasoning (i.e., the number of categories achieved using the Wisconsin Card Sorting Test), they failed to find any relationship. Unexpectedly, the group with ISA was slow in speed of finger tapping with both the dominant and non-dominant hands, but the effect was only statistically reliable in the dominant hand. Later research demonstrated that speed of finger tapping in dominant hand is highly correlated with severity of initial brain injury as measured by time to follow commands (Dikmen et al., 1995; Prigatano, 2020).

Research on the relationship of executive dysfunction and ISA after TBI has produced mixed findings. While the number of categories achieved on the WCST does not separate patients with ISA versus those with no ISA after severe TBI using the PCRS, the number of perseverative errors does appear to separate these two groups (Ciurli et al., 2011; Bivona et al., 2019). Using the Self-Awareness of Deficits Interview (SADI) in studying ISA after TBI, Bogod et al. (2003) report positive correlations with errors on the Stroop, Go–No–Go tasks and the self-order pointing test. However, in this study, overall IQ scores were also correlated with level of ISA. O’Keeffe et al. (2007) also reported that patients with TBI who demonstrate poor awareness have greater impairments in attention, executive functions, and memory. Hart et al. (2005) found that degree of ISA was associated with scores on a composite measure of executive function. Collectively, these studies also suggest that the expected correlations between behavioral markers of frontal lobe dysfunction may be attenuated in patients with TBI who also have substantial DD.

### The Measurement of Denial of Disability After Traumatic Brain Injury

Within the context of conducting intensive, holistic-oriented neuropsychological rehabilitation of postacute patients with TBI, Prigatano and Klonoff (1998) proposed that denial of disability (DD) could be distinguished from ISA. They developed a Clinician’s Rating Scale for evaluating these two separate, but often co-occurring problems. They suggested that both cognitive, affective, and behavioral features would be identified when attempting to separate ISA from DD. ISA is characterized by “cognitive perplexity” with an absence of emotional reactivity when given feedback regarding their neuropsychological impairments that they do not self-report. Moreover, patients with ISA do not attempt to provide a logical argument against what a clinician might describe as their neuropsychological impairments. In contrast, persons with DD show the opposite pattern. They attempt to mount a feasible argument as to why they are not impaired. They can become argumentative in their reactions. In addition, as a group, it was hypothesized that the patients with DD had less neuropsychological impairment in the areas of planning, initiation, self-monitoring, etc. (i.e., executive functions).

Prigatano and Klonoff (1998) had 10 items for each scale that could be checked off as indicating present or absent of features suggestive of ISA or DD. The higher the number, the more “severe” ISA or DD was thought to occur. No specific “cutoff” points were suggested nor validated by independent research. However, a score of 7 or higher would be considered severe for both dimensions based on our clinical observations.

Kortte et al. (2003) applied these rating scales to a population of 27 adults with TBI. They noted, as Prigatano and Klonoff (1998) had suggested, that ISA and DD co-occurred in their sample. Interestingly, “higher levels of denial were associated with greater use of avoidant coping strategies and greater use of these coping strategies was related to higher levels of depression” (pg. 131). They concluded that “individuals primarily in denial and individuals primarily anosognosic differ in the coping strategies they institute” (p. 131).

Terneusen et al. (Under review) recently conducted a validation study on the ISA and DD scales in a Dutch sample of 78 patients with a history of TBI. The ISA scale correlated with measures of severity of TBI (e.g., GCS vs. ISA severity, *r* = −0.32; PTA vs. ISA severity, *r* = +0.37), whereas measures of severity of initial TBI did not correlate with DD severity ratings (e.g., GCS vs. DD severity, *r* = −04; PTA vs. DD severity, *r* = +0.08). Equally interestingly, self-reported levels of anxiety using the Hospital Anxiety and Depression Scale anxiety scale had a modest *negative* correlation with DD severity (*r* = −0.22, *p* = 0.05). This is what would be expected when denial is conceptualized as a defense against anxiety.

In a qualitative study of 10 persons with moderate or severe TBI, O’Callaghan et al. (2006) found that these respondents indicated that they experienced denial when confronted with the reality that they could no longer do all the things they could do before their injuries. While they believed that denial protected them from distress, they also acknowledged that denial led to avoidant behaviors that interfered with recovery. Participants indicated that they were challenged by the effort to accept their “new selves” while still desiring to return to the “old self.”

To our knowledge, no other methods have appeared to measure DD versus ISA in persons with a history of TBI. However (Prigatano, 2012) suggested that patients who present with DD may “show greater indications of denial by their verbal responses obtained from the Thematic Apperception Test using the indicators of denial….” in the Cramer (2003) scoring system, denial is reflected “by statements of negation, blatant or clear omission of ominous components of reality when telling or relating a story, over-maximizing the positive or minimizing the negative, describing unexpected goodness, optimism, positiveness, or gentleness” (Prigatano, 2012). This approach was used in a recent study by Belchev et al. (2017). These investigators had 43 postacute patients with varying levels of severity of TBI complete the PCRS and compared their ratings on this scale with therapist ratings. Three groups were identified in their sample: overestimators of their competency using the PCRS, good estimators, and underestimators. These three groups did not differ on measures of severity of brain injury or their neuropsychological test performance with one exception. Over estimators performed significantly more poorly on the Digit Span subtest of the Wechsler Adult Intelligence Scale, IV Edition. However, both the over- and underestimators had higher denial scores than the good estimators using the Thematic Apperception Test. Level of denial, using the Thematic Apperception Test measure, did not appear to differ between the over- and underestimators, but a specific test of this difference was not reported. However, level of depression, as measured by the Beck Depression Inventory, was higher in under-estimators than in over-estimators. This basic finding has been reported in several studies (Smeets et al., 2017).

The fact that the overestimators did not have more severe brain injuries and showed no difference on the majority of neuropsychological tests compared to good and underestimators highlights the central problem that this review paper attempts to address. Without independent classification of postacute patients with TBI showing high levels of ISA (i.e., clinically significant ISA) (and low or non-existent DD) versus those showing high levels of DD (and low to non-existent ISA), the meaning of the projective test findings reported by Belchev et al. (2017) remains unclear. We would suggest that experienced clinicians independently identify a group of patients with TBI that show clinically significant ISA with minimal or low DD and compare them to patients with TBI showing clinically significant DD with minimal or low ISA on several measures. The measures should include severity of initial TBI, neuroimaging findings of brain pathology, and neuropsychological test findings (which included measures of attention/vigilance, perseverative responding, and motor speed). Independent measures of denial and ISA should also be employed. Measurements of denial may include projective techniques, structured interviews, as well as self-report questionnaires.

Livneh (2009) briefly reviews several measurement approaches that were developed primarily for studying denial associated with myocardial infarction or cancer. Studies employing these approaches often report that scores on denial measures are typically not associated with scores on depression measurements, but are related to scores suggestive of avoidance or escape strategies (p. 45). We propose that a major avoidance or escape strategy that we clinically observe is the tendency of patients to actively discourage the examining clinician from asking questions about how their TBI has influenced them.

The relatively new Illness Denial Questionnaire for patients with various medical conditions and caregivers has recently been described (Ferrario et al., 2017). That scale was standardized on 219 patients with diverse medical, including neurological illness, and 181 caregivers. Factor analytical studies of the scale suggest that three broad categories of behavior can be used to assess denial. They include minimizing or “denying” negative emotions, resistance to change, and conscious avoidance of discussing their medical condition. In light of the research with other medical conditions noted above, the conscious avoidance subscale may be especially helpful when capturing some aspects of DD in the population of persons with TBI.

### Incidence of Clinically Significant DD in Postacute Patients With a History of Moderate to Severe TBI

Since DD has not been extensively studied in persons with TBI, the incidence of clinically significant DD has not been established. We would propose, based on our clinical experience, that the incidence is at least as great as that for ISA. This may be especially true in postacute holistic oriented neuropsychological rehabilitation settings. A major methodological problem encountered when studying clinically significant DD was noted by Terneusen et al. (Under review). Some TBI patients with TBI decline to participate in research on DD because they do not want to discuss their disabilities. Some family members of these patients reported that the patients become angry if they are encouraged to participate in research on DD. As a result, persons with TBI who also have DD may be underrepresented in research. Perhaps, this problem can be addressed by studying the reasons that postacute patients with TBI are reluctant to participate in studies regarding cognitive, behavioral, or emotional problems related to their injuries.

### Neurological Correlates of DD in Patients With a History of Moderate to Severe TBI

An intriguing area of study would be the neuroimaging correlates of DD in patients with moderate to severe TBI. Since denial is considered a defense mechanism, one would not expect to find any correlation with structural brain lesions and DD in patients with TBI. However, deep brain lesions involving the basal ganglia have been associated with persistent “denial of hemiplegia” several years poststroke (House and Hodges, 1988). In addition, one single-photon emission computerized tomography (SPECT) study reported decreased activation in the caudate nucleus, putamen, and thalamus in patients with hysterical sensory motor loss (Vuilleumier et al., 2001). Quite interestingly, activation patterns returned to normal when the conversion hysteria was effectively treated. Thus, there may be neurological correlates of DD.

Finally, based on our experience with a number of patients, we would suggest that multiple body trauma associated with moderate to severe TBI may also correlated with measures of DD in this group of patients. Major orthopedic injuries produce considerable pain and often require extensive physical therapy, which can be painful. Body image is obviously affected. This is a major source of anxiety that might trigger a defensive reaction in this group of individuals.

### Neuropsychological Correlates of DD in Patients With a History of Moderate to Severe TBI

To our knowledge, there have been no neuropsychological studies relating neuropsychological test performance to DD in patients with TBI. However, there are a few papers that suggest that negative response bias on verbal learning and recognition tasks tends to occur in patients who have psychogenic seizures (Bortz et al., 1995). These patients often are described as denying various psychological conflicts in their life. In our judgment, studying negative response bias in clinically significant ISA versus clinically significant DD TBI patients may be useful.

We would also anticipate that patients with moderate to severe TBI who demonstrate high DD and low or minimal ISA would perform in the average range or have mild impairments on tests of planning, attention/vigilance, novel problem solving, preservative errors, and motor speed tests–all of which would potentially implicate frontal lobe mediated executive functions.

### Psychiatric and Psychosocial Correlates of DD in Patients With a History of Moderate to Severe TBI

Given that our approach to separating ISA from DD focuses on one being a lack of or diminished emotional state versus the other being an active (but not necessary aware) state of reducing anxiety, we suggest that patients with TBI with clinically significant DD should demonstrate a greater incidence of certain psychiatric and psychosocial histories. In this regard, we have been impressed with the histories of substance abuse, failure to finish high school or other courses of education deemed important to the individual, angry outbursts, tendency to blame others for failures at work, and an avoidance attachment style that may be prevalent in persons who demonstrate clinically significant DD after moderate to severe TBI. To our knowledge, the relationships of these variables to ISA or DD have not been studied.

### Relationship of Clinically Significant ISA and DD With Neurorehabilitation Process and Outcome After Moderate to Severe TBI

To our knowledge, no study has specifically attempted to relate DD with neurorehabilitation process or outcomes. However, studies have attempted to relate ISA to various processes and outcome variables associated with neurorehabilitation after TBI. The strength of the working alliance between a brain dysfunctional patient and therapists has been reported to relate to productivity status after TBI (Prigatano et al., 1994). Schonberger et al. (2006a) reported that higher levels of self-awareness (as measured by the PCRS) are related to stronger working alliances with the treating neuropsychologist. In another study (Schonberger et al., 2006b) measured self-awareness by comparing the patients’ and relatives’ ratings of the patients’ problems using the European Brain Injury Questionnaire. They report a strong relationship between good compliance with rehabilitation activities and good self-awareness in a mixed group of patients with TBI or cerebrovascular accident. More recent studies have supported these earlier observations. Persons with TBI with good awareness of their deficits show a greater willingness to engage in treatment (O’Callaghan et al., 2012).

Additional investigation of the relationship of DD to productivity status after TBI is needed. In this regard, it is generally recognized that patients with less severe TBI are more likely to return to work posttrauma. A very interesting study by Sela-Kaufman et al. (2013) noted that patients with TBI who showed an avoidance attachment style to parental figures early in life were less likely to return to work after rehabilitation irrespective of the severity of their TBI. This finding parallels the observations of some clinicians (Prigatano, 1999). Avoidance attachment styles may significantly correlate with the use of denial in copying with emotional difficulties.

## Conclusion and Recommendations

Our review of the literature from the past 20 years as well as our clinical experience in treating these patients indicate that ISA and DD are distinct phenomena that both pose challenges to different forms of neurorehabilitation. Treatment approaches to ISA and DD should be distinct from one another, as treatment of the patient with ISA generally involves progressively (but gently) presenting tasks to the patient, which reveal to the patient the presence and extent of a functional limitation. This should be done in a manner that encourages use of compensatory strategies (Sherer and Fleming, 2014). However, treatment of DD focuses on addressing underlying distress and defensiveness before discussion of possible functional limitations due to TBI (Prigatano, 2020). Failure to appreciate this difference can result in errors in the patient’s clinical management and engagement in the rehabilitation process. It is for this reason that the topic of how to approach the rehabilitation of a person with high ISA (or clinically significant ISA) and low or minimal DD should be clarified and compared to how to approach the rehabilitation of a person with high DD (or clinically significant DD) and low or minimal ISA. A summary of our recommendations for addressing ISA and DD is provided in Table 3.

**TABLE 3:** Different approaches to Treating/managing clinically significant ISA vs. Clinically significant DD after moderate to severe TBI.

**Treating/managing ISA**	**Treating/managing DD**
Avoid arguing with the patient regarding impaired awareness	Avoid directly confronting the patient with their denial of disability
Engage the patient in tasks the patient finds meaningful and that require skills that the patient overestimates	Avoid presenting tasks that will expose deficits the patient is currently denying
Encourage the patient to track performance on tasks and compare these performances to their self-expectations	Have the patient describe self-perceived strengths and weaknesses without associating them with TBI
Provide feedback when the patient’s performances failed to reach self-expectations or functional requirements	Engage the patient in discussions regarding their sense of self extending back before the injury and identify issues that could challenge a positive self-image
Look for opportunities to suggest compensatory strategies that will enhance the patient’s performance and praise the improved results	Discuss recent experiences the patient has found to be positive/encouraging as well as negative/threatening
Select compensatory strategies based on an understanding of organizational strategies that were used by the patient prior to injury	Engage the patient in discussions of experiences such as dreams, art, etc., that have symbolic significance, positive or negative, for the patient
A strong therapeutic alliance will enhance patient willingness to use compensatory strategies	A strong therapeutic alliance will enhance patient willingness to confront negative emotions such as anxiety and anger
Self-perceived improvements along with positive feedback from others will promote a sense of resiliency and establishment of a positive sense of self	Reduction of negative emotions and the perceived ability to manage negative emotions when they arise will promote a sense of hope and resiliency
Facilitate the patient’s continued movement toward a level of compensation and function that will result in return to a productive life with positive social engagement	Encourage the patient to leverage improved emotional control by moving forward with other activities that will increase productivity and social engagement

*TBI, traumatic brain injury; ISA, impaired self-awareness; DD, denial of disability.*

Impaired self-awareness is a direct result of a brain disorder that affects cognitive functioning as well as the patient’s ability to “subjectively experience” the extent of residual impairment and disability caused by the TBI. Consequently, no one ever “talks a patient out of their ISA.” In fact, such efforts most often are ineffective and can irritate the patient and family members while promoting stress and dissention among the treatment staff (Prigatano and Morrone-Strupiinsky, 2010). Rather, the patient has to be engaged in a variety of tasks (cognitive, physical, emotional, etc.) in which they have an opportunity to observe their behavior and determine whether their level of performance is adequate or not (Prigatano et al., 1986; Prigatano, 1999; Prigatano and Morrone-Strupiinsky, 2010). This occurs best within the context of holistic, intensive neuropsychological rehabilitation programs (Prigatano et al., 1986; Ben-Yishay and Diller, 2011). However, specific efforts at using verbal and video feedbacks have also been shown to be helpful for some patients (Schmidt et al., 2012).

When engaged in a variety of practical tasks that reveal a person’s level of competency in various domains, the patient can have periodic and momentary awareness or “online awareness” (Dockree et al., 2015) of their limitations. They can then speak with their therapist regarding the meaning of this momentary awareness of a limitation not previously noticed or remembered. If the therapist has been successful in building a therapeutic alliance with the patient, the patient now feels the need to work at using compensations to cope with the limitation in their everyday life. This only occurs, however, if the therapist can instill in the patient (via dialog and their relationship) a sense of trust in the need to use the compensation and sense of hope that compensatory strategies will help the patient in the future (Prigatano, 2020). The overall experience for the patient is one of building their competencies and sense of resiliency in dealing with life’s problems.

It should be emphasized, however, that not all patients with clinically significant ISA are capable of this type of experience. In some instances, no matter what one does, the patient simply does not “see” or experience the extent of the neuropsychological problems. Yet, some of these patients are still helped to become more independent and productive. How is this accomplished? It has been our clinical experience that this typically happens when the patient trusts either in the therapist or significant other to the point that they are willing to accept the guidance of the therapist or significant other in making decisions. As an example, a 55-year-old man who fell from a ladder while working as an electrician experienced severe ISA likely related to severe bilateral frontal lobe injuries. While he believed that he could return to work as an electrician, his wife told him to “listen to his doctors.” His doctors said it was not safe for him to go back to work as an electrician. Because he trusted his wife’s opinion so much, he followed her (and the doctors’) advice and worked in a non-technical volunteer job. He remained productive for many years despite his severe and persistent ISA.

The clinical picture is much different when the patient presents with high (or clinically significant) DD and low or minimal ISA. In this case, the patient is quick to argue that the clinician’s judgments about their level of impairment are unreliable and therefore invalid. Unlike the patient with ISA, the patient with DD often has definite and often strong emotional reactions to feedback regarding any functional limitations (Prigatano and Klonoff, 1998). A key element in approaching these patients is “to stand back” and attempt to understand their personality development and the factors that appear to be “driving” anxiety or other emotions that they do not wish to experience. Instead of giving them a series of practical tasks that may reveal a functional limitation, it is often better to initially stay away from any discussion regarding the TBI and associated disabilities. The focus should be on their perceptions of themselves in light of their personal history. In this regard, we have found it helpful to ask the patient to write out a summary of their life history to enhance our understanding of their early experiences, goals, or aspirations for the future and prior experiences that have threatened these goals. As the dialog progresses, inviting patients to discuss dreams, movies, stories, tattoos, and fairytales that have important symbolic meaning to them can reveal important features of the individual such as core conflicts (Prigatano and Salas, 2017). As a therapeutic bond or relationship development, a gradual exploration of major sources of anxiety, anger, or depression in their life can begin. This is a slow and complicated process that requires the treating clinical neuropsychologist to be an effective psychotherapist (Prigatano, 2018). It is a highly individualized process that requires a good understanding of human nature, psychodynamic features of the person, and a thorough knowledge of clinical neuropsychology (Prigatano, 2020). While there are principles that underlie this approach, application of these principles to the individual patient requires experience and skill. Success is indicated by the patient’s willingness to engage in needed rehabilitation activities as well as productive activities and social interactions. Success is also dependent on the skill of the psychotherapist to reduce the patient’s anxiety and help them recognize the need for certain rehabilitation activities.

## Limitations

The review provided here is a selective review informed by extensive clinical experience rather than an exhaustive systematic review. While a large number of articles were reviewed and the combined results summarized, many possibly relevant articles were not captured due to the limited time frame and limited number of databases searched. Study design was not graded for risk of bias, so it is possible that some low-quality evidence was included in our summaries. Our decision not to conduct a systematic review was based on our knowledge that there has only been limited study of denial of disability in persons with TBI. The limited literature on DD restricted our ability to rely heavily on evidence in drawing conclusions and recommendations. The authors judged that the combination of a limited review with information derived from years of clinical experience with these issues would be more helpful than a systematic review that found insufficient evidence to support treatment recommendations.

## Summary

This selective review paper and commentary have provided evidence that ISA and DD are different phenomena that can co-occur in patients with postacute TBI. Clinical care and research on ISA and DD after TBI will be advanced if patients with moderate and severe TBI are assessed for both ISA and DD prior to initiation of postacute clinical care and as part of participation in research protocols. Past failure to independently assess both ISA and DD has limited our understanding of DD and the co-occurrence of ISA and DD. We argue that distinguishing ISA from DD has important implications for engaging patients in the neuropsychological rehabilitation process and eventual rehabilitation outcomes. Future research should also address factors that may mediate levels of ISA and DD such as age, self-esteem, and cultural factors.

Improved assessment of DD will depend on continued development and validations of measures. At present, there are a number of approaches to assessing ISA, but only the ISA and DD scales developed by Prigatano and Klonoff (1998) have been validated for use in assessing DD after TBI. As a potential new measure, the subscale assessing conscious avoidance from the Illness Denial Questionnaire appears to hold promise. This subscale is provided in Table 4 with permission from the authors.

**TABLE 4:** Conscious Avoidance Subscale of the Illness Denial Questionnaire^1^.

**Conscious Avoidance Subscale**
□ I try to avoid thinking about this disorder/disease as much as I can.
□ I try not to pay attention to my disorder/disease.
□ I try not to speak about this disorder/disease with the doctor or other
specialists.
□ I do not want to have to look the disorder/disease in the face.
□ The less I know, the better I feel.
□ I try not to speak about this disorder/disease.
□ At times I try to convince myself that I do not have any disorder/disease.
□ The best way to cope with this disorder/disease is to not think about it.

*^1^Ferrario et al., 2017; used with permission.*

## Author Contributions

GP recognized the need for an updated review on this topic and conceived the goals for the manuscript, developed the treatment recommendations for impaired self-awareness and denial, and wrote approximately 80% of the content of the manuscript. MS developed the strategy for the abstract search and the abstract inclusion or exclusion criteria, performed substantial editing and wrote approximately 20% of the content of the manuscript, and prepared the manuscript for submission. Both authors contributed to the article and approved the submitted version.

## Conflict of Interest

The authors declare that the research was conducted in the absence of any commercial or financial relationships that could be construed as a potential conflict of interest.
